# Determination of Histamine in Silages Using Nanomaghemite Core (γ-Fe_2_O_3_)-Titanium Dioxide Shell Nanoparticles Off-Line Coupled with Ion Exchange Chromatography

**DOI:** 10.3390/ijerph13090904

**Published:** 2016-09-12

**Authors:** Natalia Cernei, Zuzana Lackova, Roman Guran, David Hynek, Jiri Skladanka, Pavel Horky, Ondrej Zitka, Vojtech Adam

**Affiliations:** 1 Department of Chemistry and Biochemistry, Mendel University in Brno, Zemedelska 1, 61300 Brno, Czech Republic; cernei.natalia3@gmail.com (N.C.); lackova14@seznam.cz (Z.L.); R.Guran@email.cz (R.G.); d.hynek@email.cz (D.H.); zitkao@seznam.cz (O.Z.); 2 Central European Institute of Technology, Brno University of Technology, Technicka 3058/10, 61600 Brno, Czech Republic; 3 Department of Animal Nutrition and Forage Production, Mendel University in Brno, Zemedelska 1, 61300 Brno, Czech Republic; sklady@mendelu.cz (J.S.); pavel.horky@mendelu.cz (P.H.)

**Keywords:** aminoacids, biogenic amines, magnetic-particles-based separation, plant, specific isolation

## Abstract

The presence of biogenic amines is a hallmark of degraded food and its products. Herein, we focused on the utilization of magnetic nanoparticles off-line coupled with ion exchange chromatography with post-column ninhydrin derivatization and Vis detection for histamine (Him) separation and detection. Primarily, we described the synthesis of magnetic nanoparticles with nanomaghemite core (γ-Fe_2_O_3_) functionalized with titanium dioxide and, then, applied these particles to specific isolation of Him. To obtain further insight into interactions between paramagnetic particles’ (PMP) surface and Him, a scanning electron microscope was employed. It was shown that binding of histamine causes an increase of relative current response of deprotonated PMPs, which confirmed formation of Him-PMPs clusters. The recovery of the isolation showed that titanium dioxide-based particles were able to bind and preconcentrate Him with recovery exceeding 90%. Finally, we successfully carried out the analyses of real samples obtained from silage. We can conclude that our modified particles are suitable for Him isolation, and thus may serve as the first isolation step of Him from biological samples, as it is demonstrated on alfalfa seed variety Tereza silage.

## 1. Introduction

**Figure 1:**
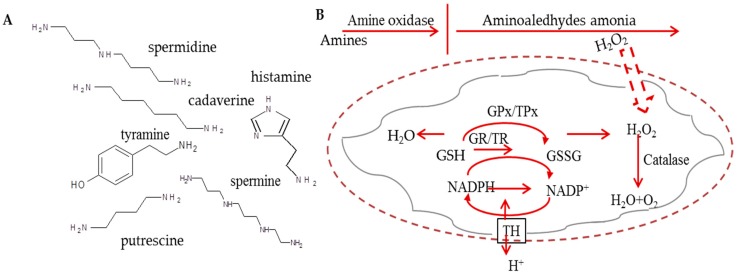
(**A**) The chemical structure of studied biogenic amines; (**B**) The scheme of amines oxidation in plants, their effects on damaging of membranes and defense mechanisms. H_2_O_2_ can be removed by catalase and HO· by mannitol. The defense mechanism of GPx/GR and TPx/TR systems and nicotinamide nucleotide TH are also shown. In the scheme, GPx stays for glutathione peroxidase; GRx for glutathione reductase; MPT for mitochondrial permeability transition; TH for transhydrogenase; TPx for thioredoxin peroxidase; and TR for thioredoxin reductase. This scheme was inspired by Agostinelli et al. [2].

The occurrence of biogenic amines (BAs) is considered as an important indicator of freshness and quality of food [1]. Therefore, it is not surprising that BAs attract an attention of various branches from chemists to food technologists. From the chemical point of view, BAs are basic, nitrogenous, low molecular-mass compounds formed as products of physiological metabolic activities of microorganisms, plants, and animals [2]. BAs can have aliphatic (e.g., spermine, spermidine, putrescine, cadaverine), heterocyclic (e.g., tryptamine, histamine), or aromatic (e.g., tyramine) structure derived mainly from the decarboxylation of amino acids [3], whereas the chemical structures of the selected BAs can be found in Figure 1A. The most amines occurring in foods originate from the corresponding amino acids, which have undergone decarboxylation by putrefactive bacteria, producing mainly putrescine (Put), Cadaverine (Cad), and histamine (Him) or lactic acid bacteria producing mainly tyramine (Tym) [4]. To produce Him, the bacteria have to express histidine decarboxylase.

From the toxicological point of view, BAs have been widely studied as potentially toxic substances, because their excessive intake was demonstrated as food poisonous [5]. Based on intensive studies, the characteristics of BAs have been identified. Him and Tym are amines with the best known deleterious psychoactive and/or vasoactive effects, while the polyamines, Put, spermidine (Spd), and spermine (Spm) are considered as constituents affecting several roles in cellular metabolism. They are involved in growth of cells and their intake in foods may be beneficial under some physiological conditions [6]. Histamine has a strong and rapid cytotoxic effect causing apoptosis induction [7], but the severity of the symptoms can vary considerably with the amount of Him ingested and an individual’s sensitivity to this compound [7,8]. The mechanism of His cytotoxicity comes from amine oxidases, which can co-regulate levels of mono- and polyamines via catalysis their oxidative deamination generating the reaction products as H_2_O_2_ and aldehyde(s) that are able to induce cell death. Particularly, H_2_O_2_ generated by oxidation reaction is able to cross the inner membrane of mitochondria and directly interacts with endogenous molecules and structures, inducing an intense oxidative stress [2], which is shown in Figure 1B.

In temperate climate regions, grass silages are produced in large quantities for feeding ruminants during winter period. However, their palatability can be decreased by some components, including BAs, mainly Him, Tyr, and Put [9]. Major issues can occur due to failure in applying good practice in processing of forages with the high protein content (e.g., alfalfa, clovers, some grasses) [10]. To improve silage preservation and guarantee the quality of this animal feed, silage additives such as chemicals, enzymes, and bacterial agents can be used [11,12]. Based on the abovementioned facts it is not surprising that several criteria for silage quality assessment are based on BA determination. Determination of BAs is usually carried out using thin-layer chromatography [13], gas chromatography, capillary zone electrophoresis [14], ion exchange chromatography (IEC) [15], enzyme immunoassay [16], and/or high-performance liquid chromatography [17,18].

In this study, we focused on the utilizing of magnetic nanoparticles off-line coupled with IEC with post-column ninhydrin derivatization and Vis detection for Him separation and detection. Magnetic separation may be employed for isolation of an analyte from a sample of complex biological matrices (food, body fluids, and plants) and may thus form the first separation and pre-concentration step for subsequent analysis [19,20,21,22]. In our previous study, we successfully developed the paramagnetic microparticles (PMPs) for isolation of broad spectra of BAs from the urine of prostate cancer patients [15]. Hence, the major aim of the presented study was synthesis and characterization of PMPs, composed of nanomaghemite (γ-Fe_2_O_3_) core, modified with titanium dioxide, which was used for magnetic isolation of Him in plant materials, particularly in silages, followed by IEC-Vis AAA 400 device (Ingos, Prague, Czech Republic).

## 2. Materials and Methods

### 2.1. Chemicals

Standards of BAs with a purity of 99% were obtained from Sigma-Aldrich (St. Louis, MO, USA). Solutions of BAs were prepared with a dilution buffer called sodium cycle, composed of 1.5 mM NaN_3_, 197 mM NaCl, and 73 mM citric acid in Milli-Q water. Furthermore, we used citric acid, sodium citrate, isopropanol, potassium hydroxide, potassium bromide, hydrochloric acid, and ninhydrin, all purchased from Sigma-Aldrich. Methyl cellosolve was purchased from Ingos (Prague, Czech Republic), as well as tin chloride. All buffer solutions were prepared with deionized water obtained using reverse osmosis Aqual 25 (Aqual s.r.o., Brno, Czech Republic). The deionized water was further purified by using a Direct-Q 3 UV Water Purification System equipped with an UV lamp (Millipore, Billerica, MA, USA). The resistance of Milli-Q H_2_O was 18 MΩ·cm^−1^. The pH was measured using a pH meter WTW inoLab (Weilheim, Germany).

### 2.2. Real Samples of Silage

The samples of silage of alfalfa seed variety Tereza were prepared without treatment (control sample). The crop comes from the site of Troubsko near Brno (270 m.a.s.l.), Czech Republic. The harvest took place 4 June 2015. Mass was ensiled 5 June 2015 and collected 8 September 2015. Samples of silage were dried at a temperature of 60 °C and milled to a particle size <1 µm prior to subsequent analysis.

### 2.3. Synthesis of Paramagnetic Microparticles

PMPs employed in this study were based on maghemite particle cores and were synthesized according to our previous study [19]. Briefly, 1 g of FeCl_3_·6H_2_O was dissolved in 80 mL of water and a solution of 0.2 g of NaBH_4_ in NH_3_ (10 mL, 3.5% w/v) was poured into the first solution with stirring. The obtained solution was heated at boiling temperature for 2 h. The obtained magnetic nanoparticles were separated by an external magnetic field and washed several times with Milli-Q water. Nanomaghemite obtained in this way was further modified as that nanomaghemite methanol water suspension was mixed with 2 mL of 97% (v/v) titanium (IV) isopropoxide. The mixture was stirred overnight using a Biosan OS-10 (Biosan, Riga, Latvia). The resulting product was separated using an external magnet and washed several times with Milli-Q water. Finally, the product was dried at 40 °C.

### 2.4. X-ray Fluorescence Analysis

X-ray fluorescence (XRF) analysis of PMPs was carried out on a Xepos (SPECTRO analytical instruments GmbH, Kleve, Germany) fitted with three detectors: Barkla scatter-aluminum oxide, Barkla scatter-HOPG, and Compton/secondary molybdenum. Analyses were conducted by the Turbo Quant cuvette method. Experimental parameters were set to measurement duration of 300 s, with a tube voltage from 24.81 to 47.72 kV, tube current from 0.55 to 1.0 mA, with a zero peak at 5000 cps and the vacuum switched off.

### 2.5. Scanning Electron Microscopy

The morphology of PMPs was characterized using an electron microscope (FEG-SEM MIRA XMU, Tescan, a.s., Brno, Czech Republic). This model is equipped with a high brightness Schottky field emitter for low noise imaging at fast scanning rates. The SEM was fitted with an Everhart Thronley type of SE detector, a high-speed YAG scintillator-based BSE detector, panchromatic CL Detector and EDX spectrometer. Samples were coated with 10 nm of carbon to prevent sample charging. A carbon coater K950X (Quorum Technologies, Grinstead, UK) was used for this purpose. Different conditions were optimized in order to reach either minimum analysis time or maximum detail during overnight automated analysis. An accelerating voltage of 15 kV and beam currents of about 1 nA gave satisfactory results regarding maximum throughput.

### 2.6. Scanning Electrochemical Microscopy

An identification of surface changes before and after biogenic amine binding to PMP surface was performed using scanning electrochemical microscope Model 920D (CH Instruments, Inc., Bee Cave, TX, USA). We used an electrochemical microscope with a 10-mm measuring platinum disc probe electrode with a potential of 0.2 V, and another platinum disc electrode with an O-ring as the conducting substrate, with a potential of 0.3 V. During scanning, the particles were attached to the substrate platinum electrode by magnetic force from a neodymium magnet, which was situated below the electrode. The platinum measuring electrode was moving from 150 µm above the surface. The scanning was carried out in a solution consisting of 5% ferrocene in methanol (w/w) mixed in a 1:1 ratio with 0.05% KCl in water (w/w). Measurements were performed in a 1.5 mL Teflon cell, according to the following parameters: amperometric mode and vertical scan was carried out in a 500 × 500 µm area with a scan rate of 30 µm·s^−1^.

### 2.7. Sample Preparation

To obtain information about behavior of our prepared PMPs, biogenic amines (250 µL) in concentrations of 100 µg·mL^−1^ were bound to them, according to isolation conditions optimized in our preliminary study dealing with amino acids [15]. For the isolation, we employed 250 µL of suspension, comprising of 1 mg·mL^−1^ PMPs in phosphate buffered saline (PBS), which were washed with Britton-Robinson buffer at pH 2 to remove undesired impurities and for activation of the surface. After the isolation, the sample was dissolved in 3 M hydrochloric acid (250 µL) and evaporated using nitrogen evaporator Ultravap RC (Porvair Sciences, Leatherhead, UK). Finally, the evaporated sample was resuspended with PBS (250 µL) and analyzed using IEC. In addition, real silage samples were prepared similarly to this protocol, with some exception found in the Results and Discussion section.

### 2.8. Ion-Exchange Chromatography

AAA 400 (Ingos, Prague, Czech Republic) IEC apparatus was used. The system consisted of a glass filling chromatographic column and steel pre-column, two chromatographic pumps for transport of elution buffers and derivatization reagent, a cooled carousel for 25 Eppendorf tubes, a dosing valve, a heat reactor, a Vis detector, and a cooled chamber for derivatization reagent. The volume of the injected sample was 100 µL with RSD 1%. We used a two-channel Vis detector with a 5 µL flow volume cuvette operated at wavelengths of 440 and 570 nm. A solution of ninhydrin was prepared in 75% methyl cellosolve (v/v) and 25% 4 M acetic buffer (v/v, pH 4.0). Tin chloride was used as a reducing reagent. Prepared solution of ninhydrin was stored under an inert atmosphere (N_2_) with cooling at 4 °C. Flow rate was 0.25 mL·min^−1^. Pressure ranged from 4.5 to 6.0 MPa. Reactor temperature was set to 120 °C. For elution, two buffers were employed: buffer A was composed of 5.5 mM C_6_H_8_O_7_, 81 mM Na_3_C_6_H_5_O_7_, 257 mM NaCl, 350 mM KBr, and 250 mL of C_3_H_8_O per 1 L of Milli-Q water, with a final pH of 5.78. For pH measurements, the WTW inoLab pH meter (Weilheim, Germany) was employed.

### 2.9. Recovery

Recovery of Him was evaluated from silage samples, spiked with an internal standard of concentration 1 mg·mL^−1^. Before isolation, 100 µL of His standard and 100 µL of water were added to silage samples. Homogenates were assayed blindly and Him concentrations were derived from the calibration curves. The spike of Him was determined as a standard measured without the presence of real sample. The calculation of recovery was performed according to Causon [23] and Bugianesi et al. [24].

### 2.10. Descriptive Statistics

Mathematical analysis of the data and their graphical interpretation were realized by Microsoft Excel^®^, Microsoft Word^®^, and Microsoft PowerPoint^®^ (Microsoft, Redmond, DC, USA). Results are expressed as mean ± standard deviation (SD), unless otherwise noted. The detection limits (three signal/noise, S/N) were calculated according to Long and Winefordner [25], whereas N was expressed as S.D. of noise determined in the signal domain unless stated otherwise.

## 3. Results and Discussion

### 3.1. Ion-Exchange Liquid Chromatography with Vis Detection of Biogenic Amines

**Figure 2:**
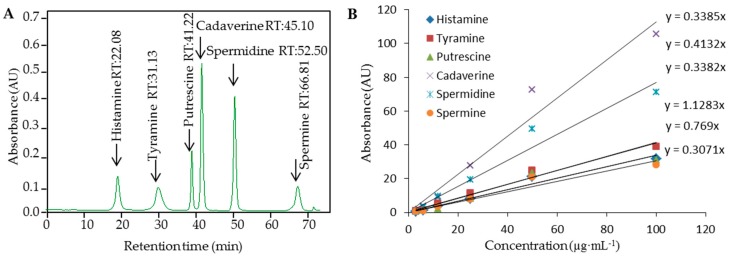
(**A**) Typical IE chromatogram of standard solution composed of selected BAs (100 µg·mL^−1^); (**B**) Calibration curves measured under the optimal conditions are shown for Histamine, Cadaverine, Tyramine, Spermindine, Spermine, and Putrescine.

Determination of Him in the silage of other plant samples is not an easy task regarding the demands of sensitivity and precision of measurements and the influences of the matrix on the sample pre-treatment steps. The conditions for BA separation and detection using IEC with ninhydrin post-column derivatization and dual channel Vis detection (λ = 570 nm, and 440 nm) were optimized in our preliminary study [15]. In this study, we attempted to combine previously found advantages of PMP-based isolation of the BAs. Based on the optimization of separation of biogenic amines, the conditions were as follows: elution of biogenic amines was performed using gradient elution with buffers of different ionic strength and pH, as well as a temperature gradient. Finally, for detection of His, the elution buffer was used (composition is mentioned in the Materials and Methods—ion-exchange chromatography section), and His was eluted under the following program: 0–45 min elution with buffer A. After separation, the column was regenerated using 0.2 mol·L^−1^ NaOH for 15 min, and stabilized for 19 min using buffer A. The column temperature was set to 76 °C. Standards of BAs, particulalry Him, Spd, Cad, Put, Spm, and Tym (100 µg·mL^−1^), measured under the optimal conditions are shown in Figure 2A. Calibration curves of BAs measured under the optimal conditions are shown in Figure 2B. Correlation coefficient equal to 0.9978 was obtained for peak area based calibration curve for determination of Him, indicating strictly linear dependencies. These values were comparable with other reported UV and fluorescence detection levels obtained using various derivatization agents [26,27].

### 3.2. PMPs-Based Isolation of BAs

**Figure 3:**
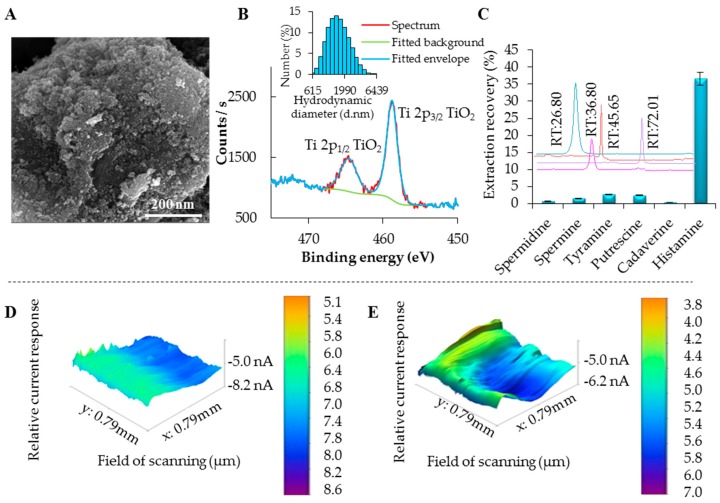
Basic characterization of prepared PMPs. (**A**) Micrograph, expressing microparticles surface and morphology, the length of scale bar is 200 nm; (**B**) XPS narrow scan of Ti2p of MAN18; (**C**) Binding specificity of the prepared particles after application of 100 µg·mL^−1^ of selected Bas. Chromatograms showing various retention times of BAs immobilized on PMPs, with expression of their peak area representing the amount specifically bound on PMPs. Scanning electrochemical microscopy 3D images characterizing the PMP electrochemical surface changes after binding of Him, relative current response of PMP surface: (**D**) for blank PMPs without Him bound to their surface and (**E**) for PMPs after binding of Him.

An issue of derivatization methods carries drawbacks such as time-consuming and laborious sample preparation, interference from by-products, long analysis times, and the risk of indeterminate errors [28,29]. For this reason, we decided to use the PMP separation approach with the ability to increase the selectivity of the isolation of the analyte and increase the sensitivity of detection. For the modification of maghemite surface, a reaction with titanium (IV) isopropoxide in methanol water solution was used. During the reaction, titanium (IV) isopropoxide is slowly hydrolyzed and titanium dioxide nanoparticles are formed on the surface of maghemite core. The morphology of resulting product is shown in SEM micrograph in Figure 3A. The presence of Ti on magnetic particles was confirmed by XRF analysis (Figure S1). Further, the particles were characterized using XPS and XRD. XPS survey spectrum is shown in Figure S2. 

Further, narrow scans of Ti2p (Figure 3B) and Fe2p (Figure S3) suggested that Ti and Fe are presented in the form of TiO_2_ and Fe^3+^, respectively. According to our calculations, TiO_2_ represents 11.6% of sample. X-ray diffraction pattern is plotted in Figure S4. See Figures S1–S4 in supplementary materials. Diffractions corresponding to the Fe_2_O_3_ substrate particles were detected only. The observed diffractions are either of maghemite or magnetite or magnetite crystal lattice, because maghemite diffractions are closer to their database positions. Selected diffractions of maghemite (PDF card no. 00-039-1346) are plotted as vertical dashed lines for comparison. No diffractions corresponding to TiO_2_ were observed, indicating either a very low amount of the TiO_2_ phase and/or its amorphous nature. The inserted diagram shows the hydrodynamic diameter of particles describing their prevailing micrometer size (Figure 3B). Titanium dioxide on the surface of maghemite is strongly bonded because maghemite has got hydroxyl groups in its structure [30]. Thus, titanium dioxide can be also covalently bonded and it leads to lowering the negative potential in comparison with unmodified maghemite. If we take into the account the presence of electron pairs on oxygens from titanium dioxide, BAs can be also bonded by hydrogen bonds.

Further, we tested the effectiveness of the prepared particles to isolate certain BAs. If one may look at the results of the extraction recovery showed in Figure 3C, Him binds to PMPs more effectively in the comparison with the rest of BAs. This can be caused by the structure of Him, especially by the presence of heterocyclic ring. Here, we can expect that some kind of coordination bond and π-π interaction can also exist [31]. Finally, extraction recoveries for various BAs isolated by PMPs MAN18, modified by titanium (IV) isopropoxide, in concentration 12 mg·mL^−1^ are shown in Table 1.

### 3.3. SECM Analysis

In the literature, it was explained that BAs are protonated under the influence of acidic conditions [27], forming the active NH_4_^+^ group [32], therefore we decided for Britton-Robinson buffer (pH 2), used for PMPs washing to remove the undesired impurities, and to protonate BA molecules providing the active sites for interaction with surface of PMPs. The surface modification using various functional groups provides different currents of bead surface, based possibly on electrostatic, hydrophobic interactions, Van der Waals forces, or hydrogen bonds between microparticles and BA molecules. The hypothesis on electrostatic interactions was suggested on the basis of our previously published results [15], where we used SECM analysis, providing information on relative current response of PMPs surface. Here, we selected the BA, which showed the largest binding affinity (i.e., Him). As it is obvious from three-dimensional (3D) images obtained from SECM analyses, the surface of PMPs without binding of analyte exhibits a positive charge in the case of PMPs modified with titanium dioxide (Figure 3D). Furthermore, we performed the isolation, and the relative current response of microparticle response was decreased to 3.8 nA for Him (Figure 3E), which confirmed our hypothesis of the best type of the particles to isolate Him, because other BAs caused negligible current changes only (not shown).

### 3.4. Real Samples Analyses

**Figure 4:**
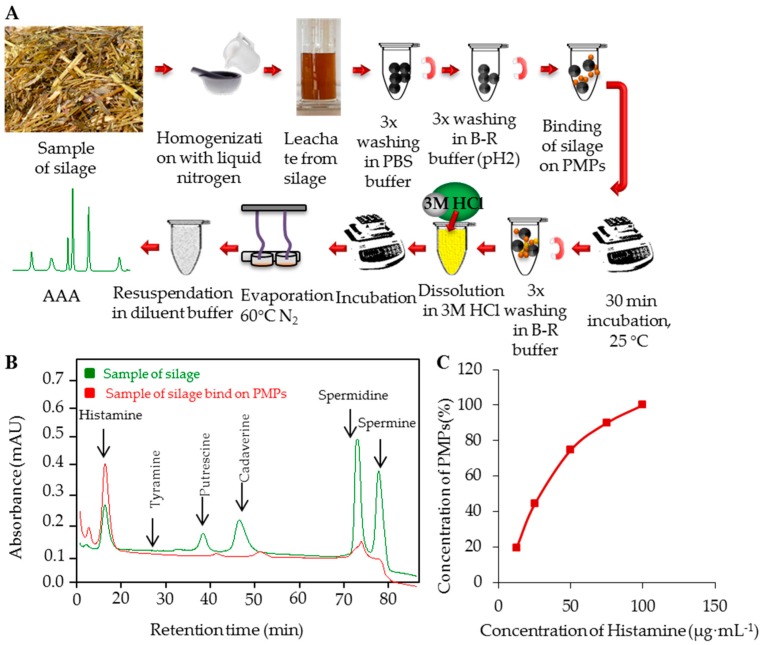
(**A**) Scheme of isolation of biogenic amines from silage sample of PMPs; (**B**) Typical chromatograms overlay: red—Samples of silage bound on PMPs and green—Sample of silage before isolation on PMPs; (**C**) Saturation curve measured under the optimal conditions within tested concentration range 2, 4, 8, 12, and 16 mg·mL^−1^ of PMPs.

Prior to the analysis of real samples, the method for the isolation of Him by PMPs MAN18 and for its detection by subsequent IEC analysis was validated. The analytical parameters of this method are shown in Table 2. To test the ability of PMPs to serve as an isolation tool applicable for analysis of real samples without pre-treatment, we decided to use samples of silage. More detailed information about silage samples (locality and concentration) is shown in Table 3. Scheme of BAs isolation from silage samples is shown in Figure 4A. Briefly, to the samples of silage (300 mg dry sample weight) 2 mL of ACS water were added. Subsequently, the samples were incubated at 37 °C, 300 rpm, 60 min in thermoblock. After the incubation, samples were centrifuged at 2500*g*, 4 °C, 20 min. Supernatants were pipetted and applied to PMPs directly (250 µL), and the optimized isolation steps according to Figure 4A were carried out.

After the incubation, Him was determined using IEC-Vis, whereas typical chromatogram overlay is shown in Figure 4B: red—sample of silage preconcentration in maximum of saturation curve, bound on PMPs, and green—sample of silage before isolation on PMPs. In all silage samples, Him was determined as the most abundant BA (15–277 µg·mL^−1^). Here, we demonstrate that the isolation process can be made directly in samples containing the analyte, eliminating the need for centrifugation or filtration [33]. For determination of the best conditions for preconcentration of BAs from the silage sample the PMP saturation curve was measured (Figure 4C). It is demonstrated that the signal height increased, i.e., the yield enhanced, with the increasing concentrations of PMPs (2–8 mg). With further increasing concentrations (16 mg), the signal is not increased due to saturation of the particle surface. Therefore, the optimal amount of PMPs was found to be 8 mg.

## 4. Conclusions

The design and performance of the method used for isolation and quantification of biogenic amines, mainly histamine, using PMP isolation was described in this study. Prepared PMPs are able to improve BA isolation from various biological matrices such as silage, as it was shown on the real samples with subsequent separation on IEC. Moreover, PMPs are well suited for utilization in fluidic devices or biosensors or Lab-on-a-Chip platforms combined with the power of magnetic field. PMPs may thus be a platform for the development of a low-cost and rapid analytical method for specific determination of histamine and elimination of the effect of interferences, such as other biogenic amines [34,35].

## Figures and Tables

**Table 1:** The recoveries of chosen biogenic amines (spermine, spermidine, tyramine, putrescine, cadaverine, histamine).

MAN18	Recovery (%)
Cadaverine	0.3
Spermidine	0.7
Spermine	1.5
Putrescine	2.4
Tyramine	2.6
Histamine	90.0

PMPs concentration 12 mg·mL^−1^ (MAN18 modified with Titanium (IV) isopropoxide).

**Table 2:** Analytical parameters of analysis of Histamine.

Compound	Mr (g·mol^−1^)	Regression Equation	Linear Dynamic Range (μg·mL^−1^)	R^2^	LOD (μg·mL^−1^)	LOQ (μg·mL^−1^)	RSD (%)
Histamine	111.2	y = 0.3234x	3.0–100.0	0.996	2	7	2.0

Mr stays for molecular mass; R^2^ for coefficient of determination; LOD for limit of detection (3 S/N); S/N means signal/noise ratio where S is the peak area of the lowest detectable signal of analyte and N is expressed as standard deviation (SD) of noise determined in the signal domain; LOQ for limit of quantification (10 S/N) and RSD for relative standard deviation calculated from three measurements of Histamine standard peak areas (100 μg·mL^−1^; injection 100 μL per sample).

**Table 3:** Histamine extraction recoveries for PMPs of concentration 2 mg·mL^−1^ and 12 mg·mL^−1^ (MAN18 modified with Titanium (IV) isopropoxide).

Date of Sampling	31 October 2015	31 October 2015	31 October 2015	25 November 2015	25 November 2015	25 November 2015	25 November 2015
Concentration of Histamine	(µg·mL^−1^)	(µg·mL^−1^)	(µg·mL^−1^)	(µg·mL^−1^)	(µg·mL^−1^)	(µg·mL^−1^)	(µg·mL^−1^)
IEC	750	420	60	43	56	42	58
PMPs (2 mg)	280	152	22	16	20	15	21
PMPs (12 mg)	690	380	54	39	50	38	52

Concentrations are expressed in µg.mL^−1^ for histamine found in real samples of silages. The crop comes from the site of Troubsko near Brno (270 m.a.s.l.), Czech Republic.

## Supplementary Materials

The following are available online at www.mdpi.com1660-4601/13/9/904/s1, Figure S1: Characterization of MAN18 using XRF, Figure S2: XPS survey scan of MAN18, Figure S3: XPS narrow scan of Fe2p of MAN18, Figure S4: XRD pattern of Fe_2_O_3_ within MAN18.
