# Supplementary material for: Determination of Histamine in Silages Using Nanomaghemite Core (γ-Fe2O3)-Titanium Dioxide Shell Nanoparticles Off-Line Coupled with Ion Exchange Chromatography
Source: Int J Environ Res Public Health. 2016 Sep 12;13(9):904. doi: 10.3390/ijerph13090904 (PMC5036737; doi:10.3390/ijerph13090904)
Supplement: Supplementary file 1 [file ijerph-13-00904-s001.pdf]

Supplementary Materials: Determination of Histamine in Silages Using Nanomaghemite Core (γ-Fe2O3)-Titanium Dioxide Shell Nanoparticles Off-Line Coupled with Ion Exchange Chromatography

Natalia Cernei, Zuzana Lackova, Roman Guran, David Hynek, Jiri Skladanka, Pavel Horky, Ondrej Zitka and Vojtech Adam

1. X-ray Diffraction Measurement

X-ray diffraction analyses were performed using SmartLab diffractometer Rigaku (Tokyo, Japan) with copper K alpha X-ray source (wavelength of 1.5406 Å). Standard powder diffraction measurements in Bragg-Brentano configuration were done.

2. X-ray Photoelectron Spectroscopy Measurement

The XPS measurements were done on the ESCALAB 250Xi Thermo Fisher Scientific (Waltham, MA, USA). An X-ray beam with power of 200 W (650 μm spot size) was used. The survey spectra were acquired with pass energy of 50 eV and resolution of 1 eV. High-resolution scans were acquired with pass energy of 20 eV and resolution of 0.1 eV. In order to compensate the charges on the surface, electron flood gun was used. Spectra were referenced to the hydrocarbon type C 1s component set at a binding energy of 284.8 eV. The spectra calibration, processing and fitting routines were done using Avantage software (Thermo Fisher Scientific, Waltham, MA, USA).

3. Figures S1–S4

The prepared nanomaghemite core (γ-Fe_2O_3)-titanium dioxide shell nanoparticles were analyzed and characterized by X-ray fluorescence (Figure S1), X-ray photoelectron spectroscopy (Figures S2 and S3) and X-ray diffraction (Figure S4).

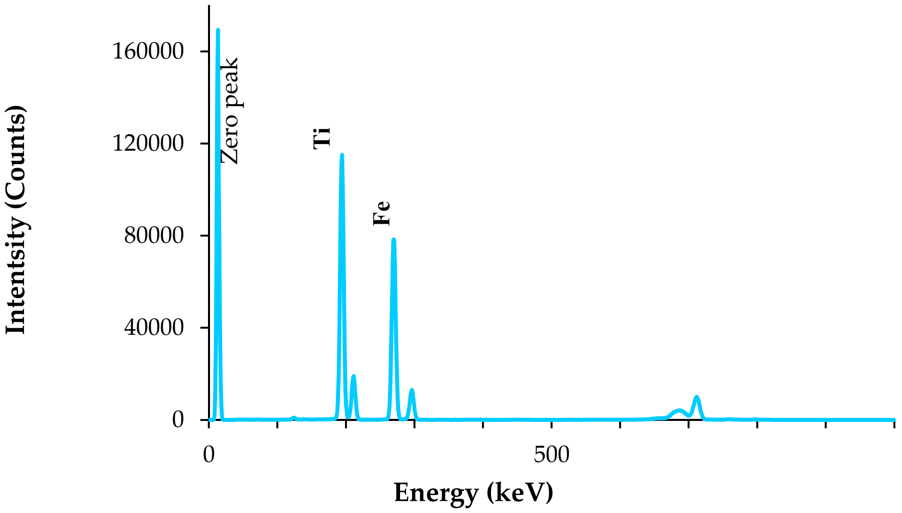

Figure S1. Characterization of MAN18 using XRF.

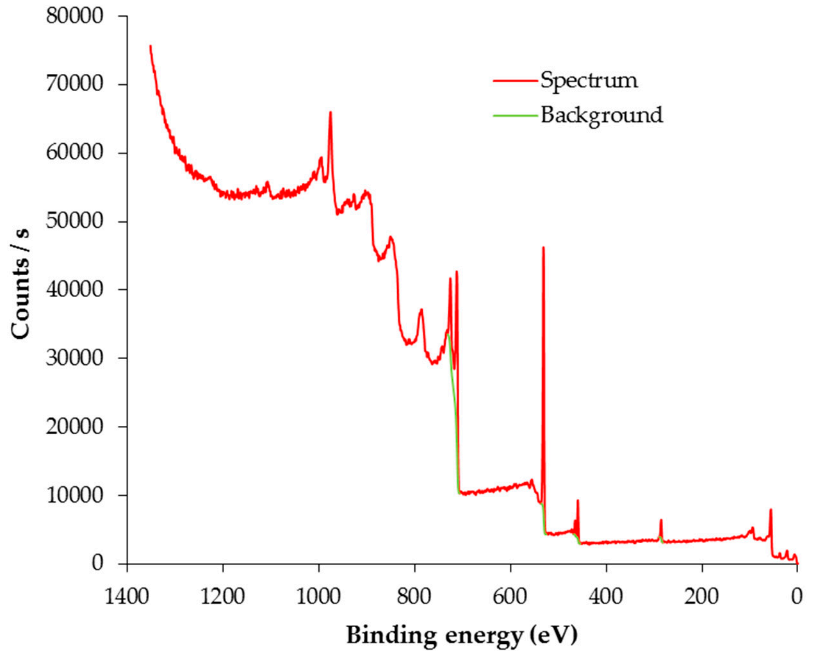

Figure S2. XPS survey scan of MAN18.

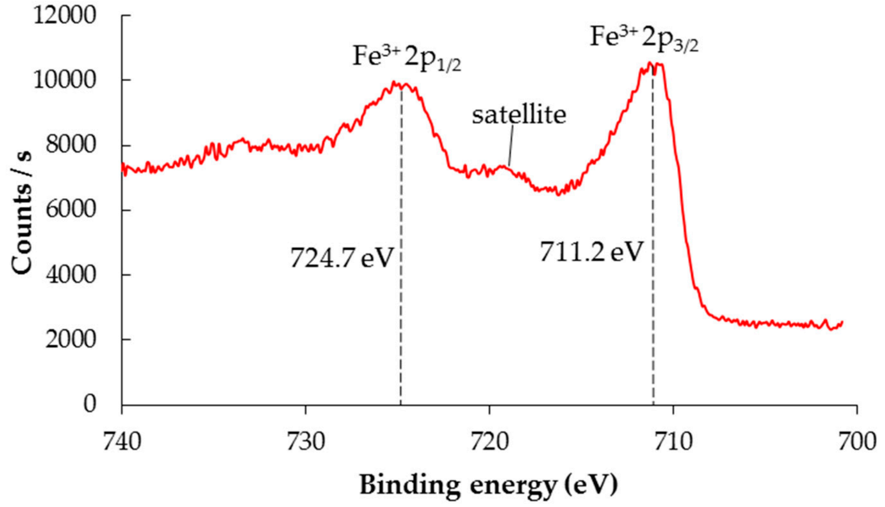

Figure S3. XPS narrow scan of Fe2p of MAN18.

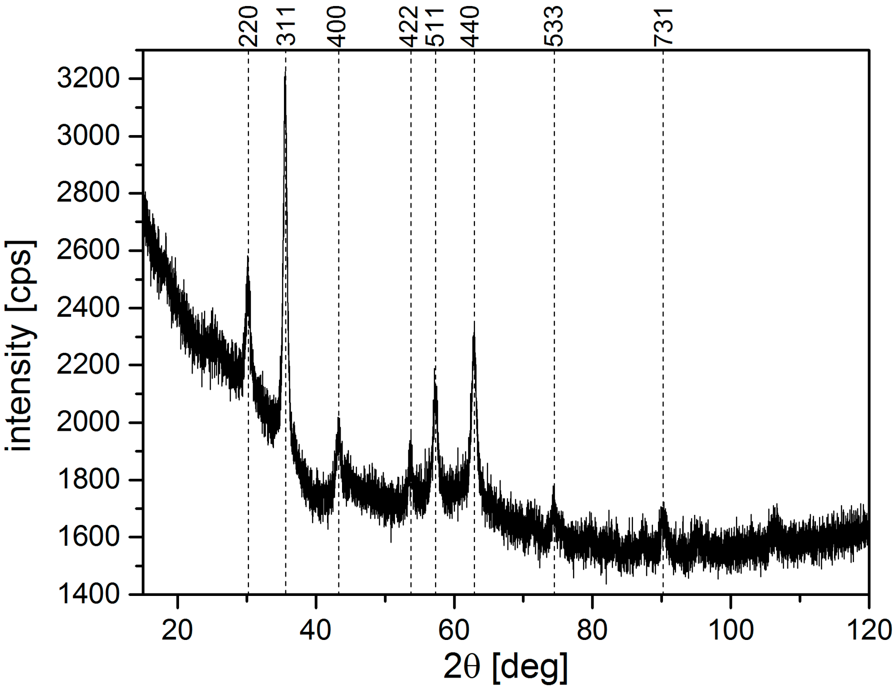

Figure S4. XRD pattern of Fe_2O_3 within MAN18.

© 2016 by the authors. Submitted for possible open access publication under the terms and conditions of the Creative Commons Attribution (CC-BY) license (http://creativecommons.org/licenses/by/4.0/).
